# Genomic analysis for heat and combined heat–drought resilience in bread wheat under field conditions

**DOI:** 10.1007/s00122-021-03969-x

**Published:** 2021-10-16

**Authors:** Michael O. Itam, Ryosuke Mega, Yasir S. A. Gorafi, Yuji Yamasaki, Izzat S. A. Tahir, Kinya Akashi, Hisashi Tsujimoto

**Affiliations:** 1 Arid Land Research Center, Tottori University, Tottori, 680-0001 Japan; 2 Graduate School of Sciences and Technology for Innovation, Yamaguchi University, Yamaguchi, 753-8515 Japan; 3 Arid Land Research Center, Tottori University, Tottori, 680-0001 Japan; 4 Agricultural Research Corporation, Wheat Research Program, P.O. Box 126, Wad Medani, Sudan; 5 Faculty of Agriculture, Tottori University, Tottori, 680-8553 Japan

## Abstract

**Key message:**

GWAS on a bread wheat panel with high D genome diversity identified novel alleles and QTLs associated with resilience to combined heat and drought stress under natural field conditions.

**Abstract:**

As heat (H) and drought stresses occur concurrently under field conditions, studying them separately offers limited opportunities for wheat improvement. Here, a wheat diversity panel containing *Aegilops tauschii* introgressions was evaluated under H and combined heat–drought (HD) stresses to identify quantitative trait loci (QTLs) associated with resilience to the stresses, and to assess the practicability of harnessing *Ae. tauschii* diversity for breeding for combined stress resilience. Using genome-wide analysis, we identified alleles and QTLs on chromosomes 3D, 5D, and 7A controlling grain yield (GY), kernel number per spike, and thousand-kernel weight, and on 3D (521–549 Mbp) controlling GY alone. A strong marker–trait association (MTA) for GY stability on chromosome 3D (508.3 Mbp) explained 20.3% of the variation. Leaf traits—canopy temperature, vegetation index, and carbon isotope composition—were controlled by five QTLs on 2D (23–96, 511–554, and 606–614 Mbp), 3D (155–171 Mbp), and 5D (407–413 Mbp); some of them were pleiotropic for GY and yield-related traits. Further analysis revealed candidate genes, including *GA20ox*, regulating GY stability, and *CaaX prenyl protease 2*, regulating canopy temperature at the flowering stage, under H and HD stresses. As genome-wide association studies under HD in field conditions are scarce, our results provide genomic landmarks for wheat breeding to improve adaptation to H and HD conditions under climate change.

**Supplementary Information:**

The online version contains supplementary material available at 10.1007/s00122-021-03969-x.

## Introduction

In many wheat-growing regions, heatwaves and drought episodes occur concurrently and are considered the most damaging climatic stressors for wheat (Zampieri et al. 2017). In the current climate change scenario, every 1 °C rise in global mean temperature results in a 6% reduction in wheat yield, and a 17% increase in agricultural water supply is needed to prevent drought stress (Pennisi 2008; Zhao et al. 2017). This implies that global wheat production will continue to be lower than demand, especially as global population increases (Food and Agriculture Organization of the United Nations 2020). In semiarid regions, where heatwaves and drought episodes are common, an annual grain yield (GY) increase of up to 2.7% is needed (Iizumi et al. 2021). Such an increase may be difficult to achieve using the current elite germplasm, which has a narrow gene pool (Ogbonnaya et al. 2013). Therefore, the use of new genetic resources has the potential to facilitate wheat breeding for resilience to combined stresses (Reynolds et al. 2015).

Wheat wild relatives are a good source for developing new genetic materials; one such relative is *Aegilops tauschii*, the D genome progenitor of bread wheat (Tsujimoto et al. 2015). A wheat multiple synthetic derivative (MSD) population was developed using 43 *Ae. tauschii* accessions as a platform to explore the genetic diversity of *Ae. tauschii* for wheat improvement (Tsujimoto et al. 2015; Gorafi et al. 2018). This population exhibited high genetic diversity when characterized under heat (H) stress in Sudan (Elbashir et al. 2017). Under drought stress in Japan, some MSD lines showed better adaptation than their backcross parent and check cultivars (Itam et al. 2020a, 2021). However, the genetic basis of the diversity in resilience to H, drought, and combined heat–drought (HD) stress has not been fully explored. Moreover, reports on genome-wide association studies (GWAS) for HD in bread wheat under natural field conditions are scarce. Qaseem et al. (2019) and Schmidt et al. (2020) reported shared genomic regions across different conditions, including HD stress, in wheat cultivars and landraces grown in polytunnels. A few other GWAS conducted under similar or more controlled environments have been extensively reviewed (Tricker et al. 2018). However, results from controlled environments may not be replicated in natural field conditions, and information from field conditions is needed to apply the findings to practical breeding.

The objective of this study was to identify QTLs associated with H and HD stress resilience in bread wheat under field conditions and to assess the practicability of harnessing *Ae. tauschii* diversity for combined stress resilience breeding. We evaluated a systematically selected wheat diversity panel (consisting of 145 MSD lines) under H and HD in Wad Medani, Sudan, in 2019 and 2020, and identified novel alleles and QTLs for several traits, including GY and related traits. The loci for most leaf traits, including canopy temperature and normalized difference vegetation index (NDVI), were pleiotropic for GY and related traits. The identified candidate genes suggest the role of gibberellin homeostasis in maintaining GY stability and of CaaX prenylation in regulating canopy temperature under the combined stress. Our study provides new genetic materials and QTLs for breeding wheat with improved resilience to H and HD conditions.

## Materials and methods

### Plant materials

A diversity panel of 145 MSD lines and 5 check cultivars was used (Table S1). The study was originally designed with 155 MSD lines and 5 check cultivars, but 10 MSD lines did not flower due to vernalization requirement and were excluded. The 145 lines used are a subset of 400 MSD lines characterized for H tolerance in Sudan (Elbashir et al. 2017). The MSD panel contained introgressions from 37 accessions of *Ae. tauschii* (DD genome) and the durum wheat cultivar ‘Langdon’ (AABB genome) (Matsuoka and Nasuda 2004). The *Ae. tauschii* accessions used were originally collected from HD stress–prone areas in the Middle East and Central Asia, including China and the Caucasus. The resulting synthetic hexaploid lines (AABBDD) were crossed with the Japanese bread wheat cultivar ‘Norin 61’ (hereafter N61, AABBDD). To reduce linkage drag, the F_1_ hybrids were backcrossed to N61 (Tsujimoto et al. 2015). Therefore, the A and B genomes of the MSD lines are biparental (from ‘Langdon’ and N61), whereas the D genome is multiparental (from 37 *Ae. tauschii* accessions and N61). The lines in the diversity panel were selected based on similar days to 50% heading (DH) and were evaluated under H and HD stress in Sudan during the 2018–19 (BC_1_F_6_ generation) and 2019–20 (BC_1_F_7_ generation) growing seasons (hereafter 2019 and 2020 seasons, respectively).

### Experimental site and design

All experiments were conducted at the Gezira Research Farm, Agricultural Research Corporation, Wad Medani, Sudan (14°24′N, 33°29′E, 407 m above the sea level). The Gezira Research Farm is a dry, hot irrigated field categorized in mega-environment 5 for wheat cultivation (Gbegbelegbe et al. 2017). It has a heavy clay soil (pH 8.0–8.4) with low contents of organic matter (< 5%), nitrogen, and phosphorus (Elbashir et al. 2017). Each experiment was designed in an alpha lattice with two replications. A total of 8 blocks per replication with 20 plots per block were used. Each plot had four rows, 1 m long and 0.2 m apart.

### Field management and drought treatment

**Fig. 1:**
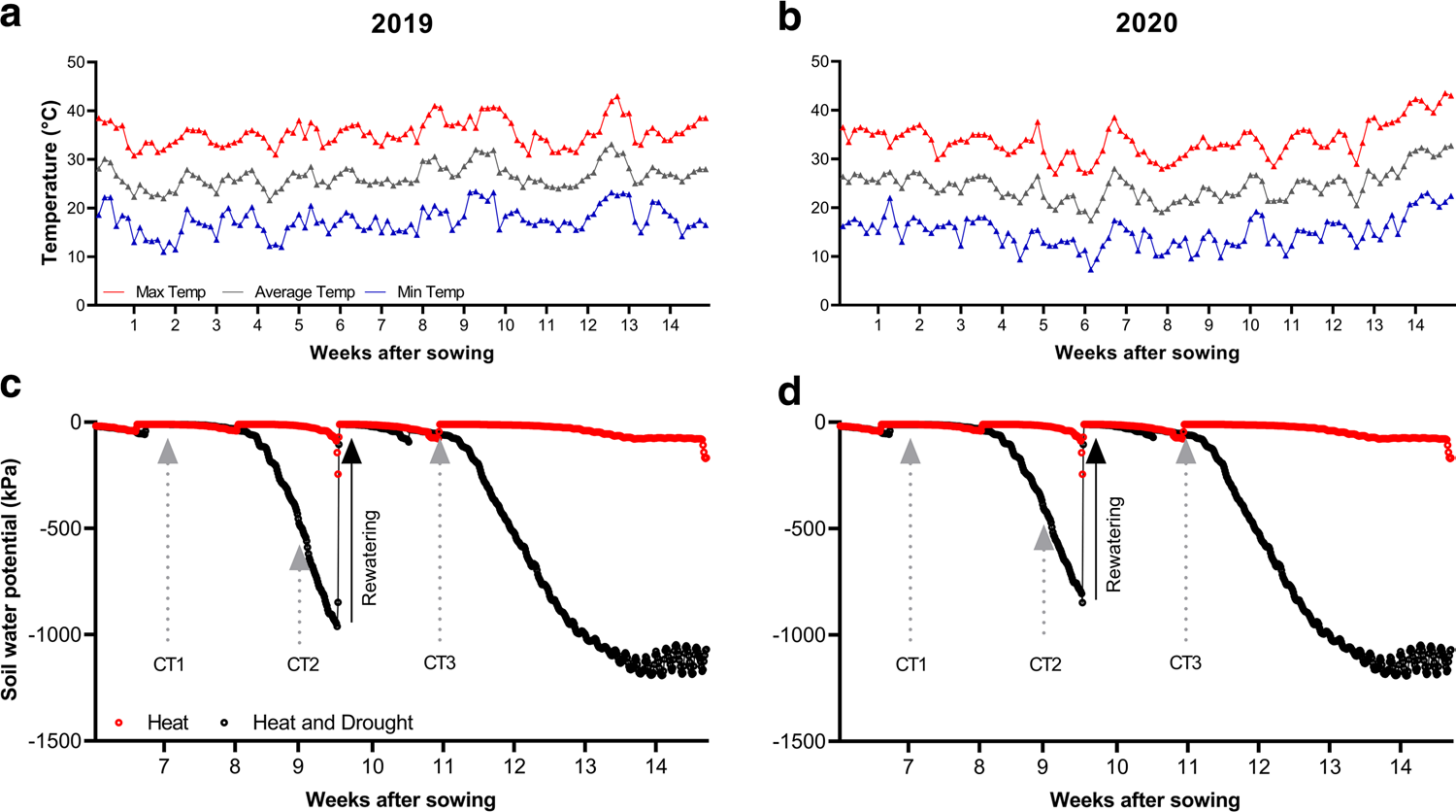
Field conditions. **a**, **b** Daily air temperature in **a** 2019 and **b** 2020. **c**, **d** Soil water potential in heat and combined heat–drought conditions in **c** 2019 and **d** 2020. The dotted arrows indicate the time points when the three canopy temperature measurements (CT1, CT2, and CT3) were taken

Seeds were treated with the insecticide Gaucho (imidacloprid, 35% WP, Bayer Crop Science, Kansas City, MO, USA) at 0.75 g kg^–1^ seed to control insect pests. The treated seeds were manually sown at 120 kg ha^–1^ during the last week of November. Field management and drought treatment were as described in Elhadi et al. (2021). Before sowing, phosphorus was applied as superphosphate by furrow placement at a rate of 43 kg ha^–1^ of P_2_O_5_. Irrigation was applied every 10–12 days, and the plots were hand-weeded at least twice. Soil water potential was monitored every 2 h by sensors (Decagon Devices, Pullman, WA, USA) buried 20 cm in the soil. To create the HD condition, drought was imposed by withholding irrigation when 50% of all genotypes had reached flowering, while regular irrigation continued under the H condition. To avoid permanent wilting, plots under the HD condition were re-watered when the soil water potential approached − 900 kPa (Fig. 1). In Wad Medani, Sudan, there is no rain during the winter season and the relative humidity is generally low (Elsheikh et al. 2015). We obtained relative humidity data from January to March 2020, and the daytime value was between 20 and 30%. The air temperature and relative humidity of the field were obtained from the Sudan Meteorological Agency and from a weather station within the field.

## Evaluated traits

### Morphophysiological traits

Morphophysiological traits were measured according to Pask et al. (2012). Chlorophyll content (SPAD), ground cover (GC), and NDVI were measured during the grain-filling stage. The SPAD readings were taken from the center of three randomly selected flag leaves per plot using the Minolta SPAD-502 chlorophyll meter (Konica-Minolta, Japan). The GC was estimated using a visual scale of 0–10, with 0 corresponding to 0% cover and 10 to 100% cover. NDVI was measured from plant canopy using a handheld optical sensor (GreenSeeker, Trimble Inc., Sunnyvale, CA, USA), and the measurements were taken 50 cm above the middle of each plot. Canopy temperature (CT) was measured three times: at 7 days before flowering (CT1), during flowering (CT2), and during grain filling (CT3). The CT readings were taken from the canopy of each plot on clear, calm afternoons (between 13:00 and 14:00) using a handheld infrared thermometer (Everest Interscience, Tucson, AZ, USA). The thermometer was angled with the field of view avoiding any bare soil between rows. Plant height (PH), biomass (BIO), number of spikes per plot (SN), number of kernels per spike (KPS), GY, thousand-kernel weight (TKW), and harvest index (HI) were determined at maturity. PH was measured from the soil surface to the top of the spikes excluding awns. BIO was measured as above-ground dry weight per plot. Ten randomly sampled spikes were used to estimate KPS. GY was determined as grain weight per plot. TKW was determined from the weight of 200 randomly sampled grains. The DH was recorded when 50% of the spikes in a plot had headed. The days to physiological maturity (DM) was recorded when 50% of the spikes in a plot showed total loss of green color, while the number of days from heading to maturity was recorded as grain-filling duration (GFD).

### ^*13*^*C isotope (δ*^*13*^*C) analysis*

The ^13^C composition of flag leaves was analyzed using an elemental analyzer connected to a continuous-flow isotope ratio mass spectrometer (EA/IRMS; Thermo Fisher Scientific) as described in Itam et al. (2020b). The analysis was conducted in the laboratory of Arid Land Research Center, Tottori University, Japan. Dry flag leaf samples were collected from three or more randomly selected plants per plot. The flag leaf samples (1 mg) were put into tin capsules (5 mm × 9 mm, Lüdi Swiss, Switzerland) and entered into a combustion oven by an autosampler. Each sample was measured against CO_2_ calibrated with an isotope standard to an accuracy of ± 0.066‰ SD. Finally, the ^13^C composition was calculated as δ^13^C = [(*R*_sample_/*R*_standard_) − 1] × 1000, where *R* is the ^13^C/^12^C isotope ratio.

### Genotyping-by-sequencing for association mapping

Total genomic DNA was extracted from leaves using the CTAB method (Saghai-Maroof et al. 1984), and DNA samples (20 μl; 50–100 ng μl^–1^) were sent to Diversity Arrays Technology (DArT) Pty. Ltd., Australia (http://www.diversityarrays.com) for whole-genome scanning using DArT-seq markers. Complexity was reduced using a combination of restriction enzymes to obtain a subset of restriction fragments for each sample (Sansaloni et al. 2011). The restriction fragments were then sequenced and aligned to the wheat_Chrom_Wheat_Norin61_v1.1 reference genome. The nucleotide polymorphisms (referred to as SNP markers) present in the restriction fragments were used for GWAS. The SNP markers are codominant in nature and were scored “0” (homozygous reference allele), “1” (homozygous SNP allele), or “2” (heterozygote). The markers were filtered on the basis of minimum reproducibility (95%), call rate (95%), and average read depth. A total of 14,382 SNP markers were used for association mapping. The A, B, and D genomes contain 5965 (41.4%), 6074 (42%), and 2343 (16.2%) markers, respectively.

## Statistical analysis

Best linear unbiased estimates (BLUEs) were obtained for each trait under H and HD using the residual maximum likelihood method implemented in META-R (Alvarado et al. 2020). In BLUEs, genotypes and environments were considered as fixed factors, while replication and block were random factors. To minimize the possible confounding effect of heading date, another set of BLUEs (adjusted BLUEs) was calculated by using DH as a covariate (Sukumaran et al. 2018). To separate drought response (DR) from H response in the HD condition, we divided the predicted means under HD by those under H for all traits except days to heading (Schmidt et al. 2020). Analysis of variance was performed for all evaluated traits in GenStat 19th edition (http://www.genstat.co.uk). Broad-sense heritability was estimated for each trait in Plant Breeding Tools v. 1.3 (http://bbi.irri.org). To assess genotype stability across different conditions, GY stability index was calculated using the Finlay–Wilkinson regression model: *y*_*ij*_ = μ + *G*_*i*_ + β_*i*_*E*_*j*_ + ε_*ij*_, where the regressand *y*_*ij*_ is the mean GY of the genotype, the intercept μ + *G*_*i*_ corresponds to the genetic main effect, the slope β_*i*_ corresponds to genotype variability in GY across environments (i.e., GY stability index), the regressor *E*_*j*_ is the population-wide variability in GY across environments (i.e., environmental index), and ε_*ij*_ is the error term (Finlay and Wilkinson 1963).

### Genome-wide association study and candidate gene analysis

Genome-wide association analysis was performed with both adjusted and unadjusted BLUEs for H, HD, and DR in 2019 and 2020, using the generalized linear model and mixed linear model implemented in TASSEL v. 5 (Bradbury et al. 2007). The generalized linear model was fitted with the first five principal components, whereas the mixed linear model was fitted with the principal components and the Centered_IBS kinship matrix (Yu et al. 2006). The best-fit model for each dataset was selected using quantile–quantile plots. Significant marker–trait associations (MTAs) were determined at a threshold of − log(*p*) = 3, and the quantile–quantile and Manhattan plots were generated in the R package “qqman” (Turner 2018). Then, the *p*-values were adjusted for multiple testing using false discovery rate (FDR) (Benjamini and Hochberg 1995) at the 0.05 and 0.2 thresholds. To better explore the contribution of the *Ae. tauschii* D genome, we also conducted a D genome–wide analysis using only the D genome markers. Because the A and B genomes in this panel are biparental, whereas the D genome is multiparental, conducting an additional GWAS on the D genome increased the statistical power of the analysis. The same analysis method was used for both GWAS. Stable MTAs were found in two or more conditions, and pleiotropic MTAs were found for two or more traits.

The linkage disequilibrium (LD) of the markers was calculated from observed and expected allele frequencies using TASSEL. To determine a critical point beyond which LD is due to true genetic linkage, r^2^ values of the unlinked markers (markers on different chromosomes) were explored. These values were square root transformed, and their 95th percentile was taken as the critical point. Polynomial regression-based curves were fitted on scatter plots of r^2^ against marker distance, and the critical r^2^ was then used to estimate the LD decay (Breseghello and Sorrells 2006).

To identify candidate genes, we conducted a BLASTN search of the stable and pleiotropic MTAs that passed the FDR 0.05 threshold against the IWGSC RefSeq v. 1.0 (https://urgi.versailles.inra.fr/blast/). The genes within 1 Mbp of each SNP flanking region were screened based on the literature, and eight candidate genes were selected. Candidate gene annotations were obtained from Ensembl Plants release 103 (Howe et al. 2021). The expression patterns of two candidate genes, one controlling GY, GY stability, and NDVI, and another controlling CT3, were investigated in the Genevestigator software (www.genevestigator.com, dataset: mRNA-Seq Gene Level *Triticum aestivum,* ref: IWGSCv1.1).

## Results

### Field conditions in 2019 and 2020

The average air temperature at the Gezira Research Farm ranged from 21.6 to 33.1 °C in 2019 and from 17.4 to 28.0 °C in 2020. The maximum temperature ranged from 30.8 to 43.0 °C in 2019 and from 27.0 to 43.5 °C in 2020. As expected, the highest air temperatures were recorded toward the end of the growing season which corresponds to the reproductive stages in both years (Fig. 1a, b). Soil water potential decreased from near 0 before drought stress to near − 900 kPa during severe HD stress in both seasons (Fig. 1c and d).

### Effect of heat and combined heat–drought on trait variability under field conditions

The genotype-by-environment (G × E) interaction effects were significant for DH, GFD, GY, KPS, SPAD, and TKW in 2019, and for DM, GY, HI, and KPS in 2020 (Table S2). DH, HI, and PH had consistently higher heritability values (ranging from 0.70 to 0.89) than other traits, whereas BIO, CT1, CT2, CT3, SN, and SPAD had relatively low heritability (Table 1). Under HD condition, CT2 had moderate heritability, indicating a significant genetic control, whereas under H condition heritability was low, indicating low genetic control (Table 1). Except for CT2 in 2019, mean CT values tended to be lower under HD than under H condition. The DM, GFD, HI, and TKW values were also lower under HD than under H condition in both years. Consequently, GY was lower under HD than under H, indicating a more severe effect of the HD condition (Table 1). The mean GY was 2735 kg ha^–1^ under H and 1588 kg ha^–1^ under HD in 2019, and 3116 and 2297 kg ha^–1^, respectively, in 2020, indicating higher performance (*p* < 0.05) in 2020.

**Table 1:** Descriptive statistics and heritability (*h*^2^) estimates of the bread wheat panel under heat and combined heat–drought stress in 2019 and 2020 and predicted means of ‘Norin 61’ (the backcross parent of the MSD lines) and ‘Imam’ (a popular Sudanese cultivar)

Trait	H2019				H2020					HD2019				HD2020				
	Range	Mean	N61	Imam	Range	Mean	N61	Imam	*h*^2^	Range	Mean	N61	Imam	Range	Mean	N61	Imam	*h*^2^
BIO	4312–13,812	9180	11,625	10,125	4456–16,926	10,103	10,017	12,339	0.30	2125–11,125	6292	5625	7062	2798–15,196	8682	8204	10,526	0.2
CT1	18.47–30.49	28.01	29.25	25.9	19.00–30.24	23.04	21.08	20.12	0.00	22.16–32.76	27.75	27.75	29.35	18.50–27.58	22.02	22.03	19.52	0.32
CT2	15.85–25.4	20.84	18.15	19.55	23.42–29.66	26.07	25.91	25.17	0.00	19.4–26.15	22.67	21.70	21.75	20.52–26.27	23.53	25.46	21.42	0.45
CT3	22.94–35.41	27.15	27.17	24.77	23.34–29.46	26.04	26.45	24.01	0.33	22.82–29.57	25.59	26.72	25.52	21.7–29.84	23.99	25.15	24.01	0.13
DH	50–64	58	59	64	53–72	61.71	58	64	0.70	51–65	57	58	63	49–74	63	57	68	0.77
DM	80–90	87	87	90	87–108	98	89	105	0.66	74–91	84	83	89	85–106	95	89	96	0.59
GC	2.25–4.50	3.60	3.75	3.25	NA	NA	NA	NA	NA	1.75–4.25	3.32	3.75	3.25	NA	NA	NA	NA	NA
GFD	23–38	29	29	26	29–50	36	31	41	0.32	23–37	27	25	26	24–42	32	32	28	0.75
GY	1032–5094	2735	2626	3594	1171–4687	3116	3735	4586	0.62	469–3706	1588	1375	1775	408–4014	2297	3358	2861	0.44
HI	8.75–42.75	30.07	22.40	35.60	16.9–45.46	31.32	40.22	36.14	0.77	8.17–41.84	25.45	24.49	25.89	11.73–48.07	26.74	44.46	26.57	0.63
KPS	17–47	29	27	34	10–49	29	29	42	0.64	14–42	29	25	23	4–51	30	30	45	0.58
NDVI	0.30–0.74	0.57	0.57	0.63	0.43–0.74	0.65	0.66	0.66	0.29	0.2–0.65	0.46	0.43	0.56	0.50–0.73	0.64	0.63	0.69	0.59
PH	57.1–94.0	73.9	78.5	75.0	65.3–106.8	87.5	84.6	88.1	0.89	53.0–90.0	69.0	67.5	65.0	62.6–103.2	84.8	83.8	79.8	0.82
SN	200–515	301	275	440	223–712	488	473	662	0.18	305–620	414	500	495	219–707	437	328	707	0
SPAD	42.55–56.00	49.52	48.90	49.55	19.94–44.96	34.49	37.44	36.82	0.00	43.95–56.30	49.77	45.80	49.20	16.09–45.64	32.87	35.09	30.37	0
TKW	20.2–45.4	30.1	32.2	36.8	20.3–49.3	34.2	34.7	28.6	0.56	16.4–37.1	25.2	23.1	28.8	20.9–41.4	31.9	28.2	30.1	0.42
δ^13^C	− 29.97–( − 26.89)	− 28.28	− 28.32	− 29.32	NA	NA	NA	NA	NA	− 29.07–( − 26.28)	− 27.70	− 27.86	− 27.87	NA	NA	NA	NA	NA

BIO, biomass; CT1, canopy temperature 7 days before flowering; CT2, canopy temperature at flowering; CT3, canopy temperature at grain filling; DH, days to 50% heading; DM, days to maturity; GC, ground cover; GFD, grain-filling duration; GY, grain yield; HI, harvest index; KPS, kernel number per spike; NDVI, normalized difference vegetation index; PH, plant height; SN, number of spikes per plot; SPAD, chlorophyll content; TKW, thousand-kernel weight; δ^13^C, delta carbon-13 value

DH was used as a covariate and, hence, it is not shown in the coefficient of variation (CV)

GY correlated significantly (*p* < 0.05) with most of the evaluated traits in both conditions in both years (Table S3). The GY–BIO and GY–HI correlations were consistently high, with correlation coefficients (*r*) ranging from 0.564 to 0.742 (*p* < 0.01) in both conditions in both years. In contrast, all CT values tended to correlate negatively with most traits, including GY and BIO, under H in 2019 and under both conditions in 2020 (Table S3). Similarly, δ^13^C was negatively correlated with GY in both conditions, and with BIO, HI, and SPAD under HD in 2019. DH and GFD were negatively correlated in both conditions in both years (Table S3). We found low positive correlations between the two conditions in most traits except DH and PH, which had high correlations (*r* = 0.619–0.806, Table S3), likely because the combined stress was imposed after heading.

**Fig. 2:**
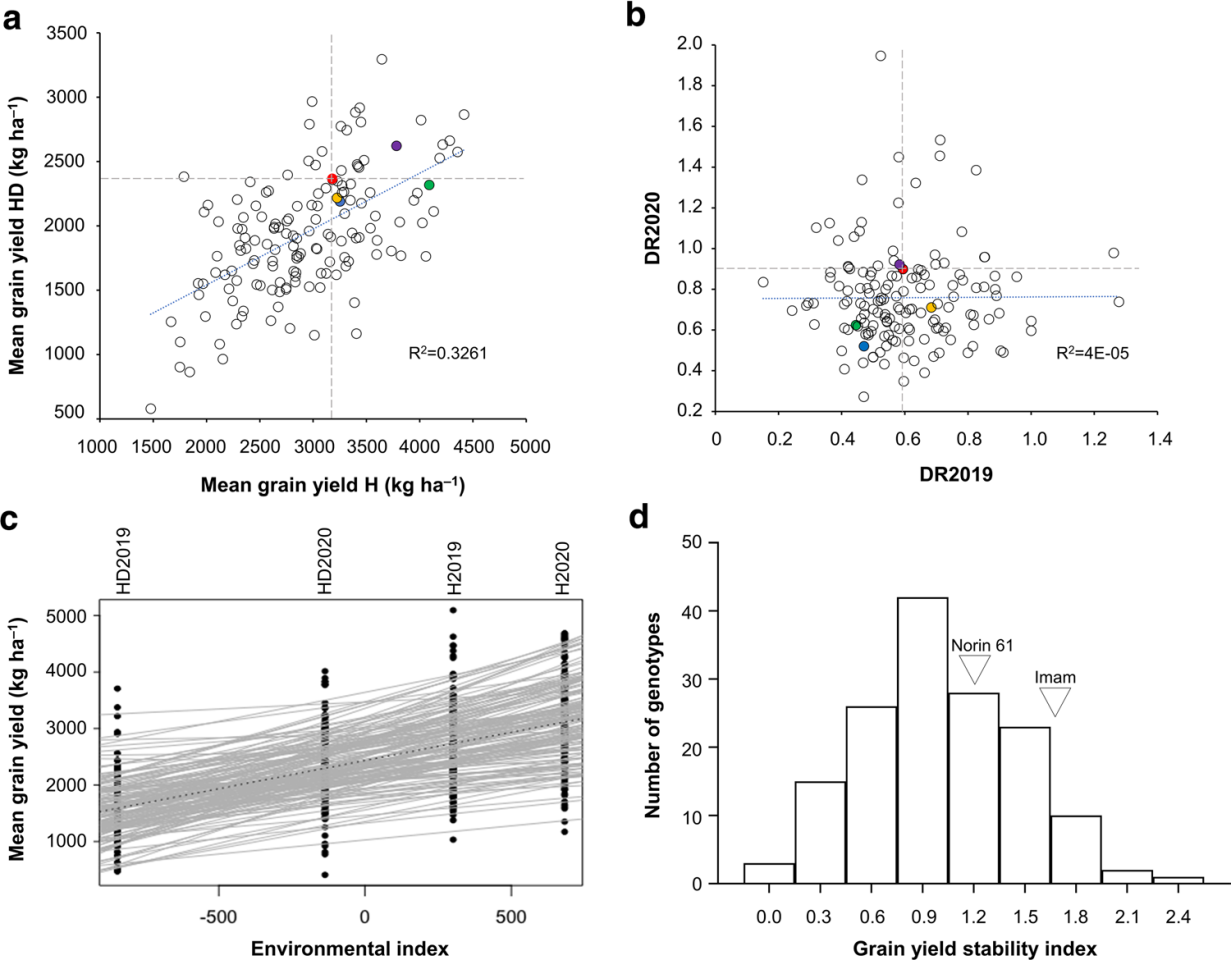
Grain yield parameters of the investigated genotypes. **a** Average grain yield under heat (H) and heat–drought (HD) conditions, (b) drought response under heat stress (DR) in 2019 and 2020. Dashed gray lines intersect on ‘Norin 61’ (red circle), the backcross parent. Check cultivars: green circles, ‘Imam’; blue circles, ‘Fielder’; violet circles, ‘Roelf’; and yellow circles, ‘Gomria’ (**c**, **d**) yield stability index across the four environments. **c** Each line represents mean grain yield for each genotype. The dashed line represents the population mean. Most of the genotypes showed a decreasing trend in grain yield under HD. **d** The most stable lines have a lower stability index (< 1.0) compared with the less stable lines. Some genotypes are more stable than ‘Norin 61’ and ‘Imam’

Some MSD lines had higher average yield performance under H and HD conditions than the check cultivars, indicating considerable genetic gains (Fig. 2a). Some MSD lines had higher DR values than the check cultivars in both years (Fig. 2b). The decreasing yield trend under HD conditions is shown in Fig. 2c. Yield stability index was higher in some MSD lines than in the check cultivars, including N61 and ‘Imam’ (Fig. 2d). Lines with high GY and considerable GY stability are listed in Table S4.

### MTAs

**Fig. 3:**
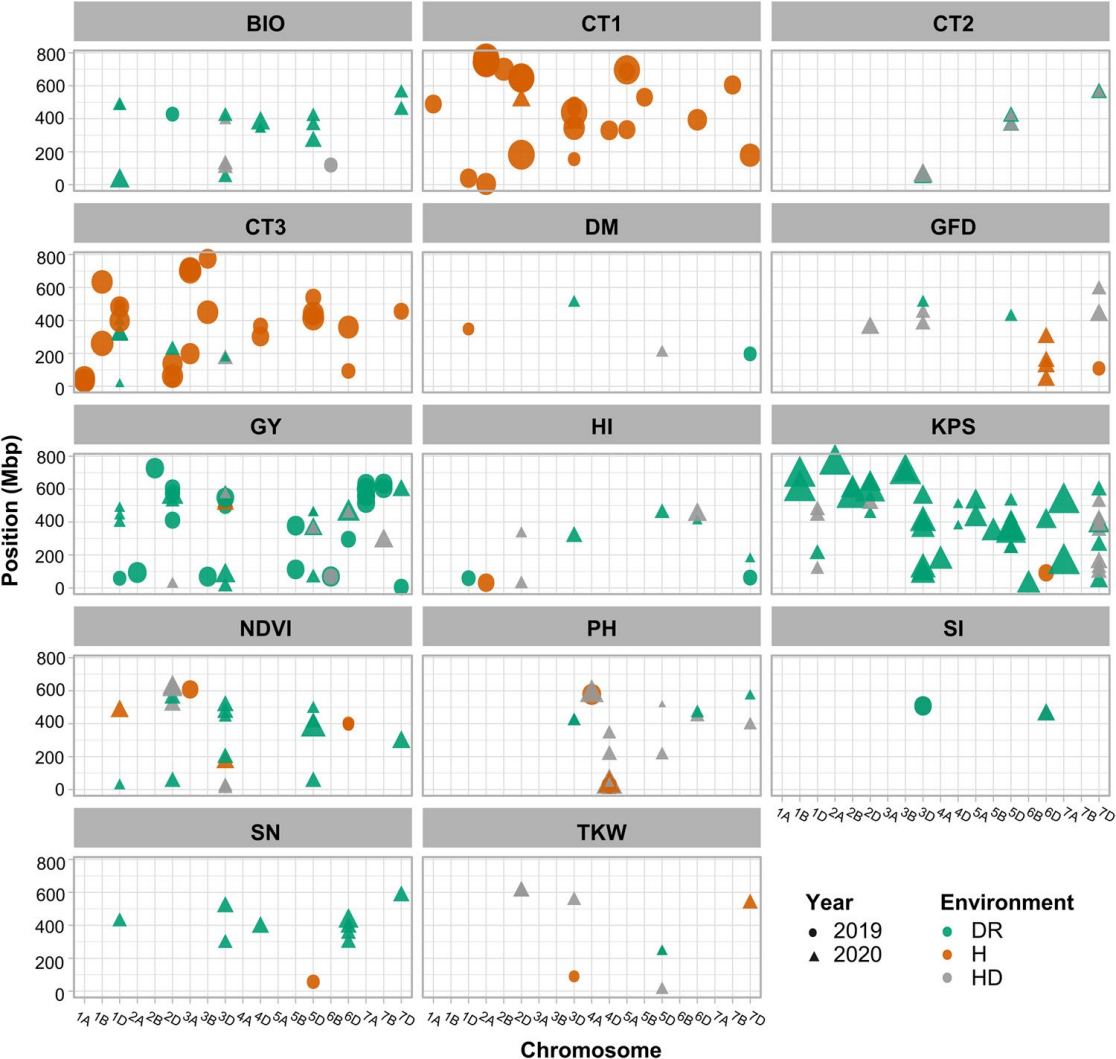
Physical positions of markers associated with evaluated traits under heat (H) and combined heat–drought (HD) conditions, and in the drought response under heat stress (DR). Symbol size corresponds to the allelic effect of each MTA. BIO, biomass; CT3, canopy temperature at grain filling; DM, days to maturity; GFD, grain-filling duration; GY, grain yield; HI, harvest index; KPS, kernel number per spike; NDVI, normalized difference vegetation index; PH, plant height; SI, grain yield stability index; TKW, thousand-kernel weight

**Fig. 4:**
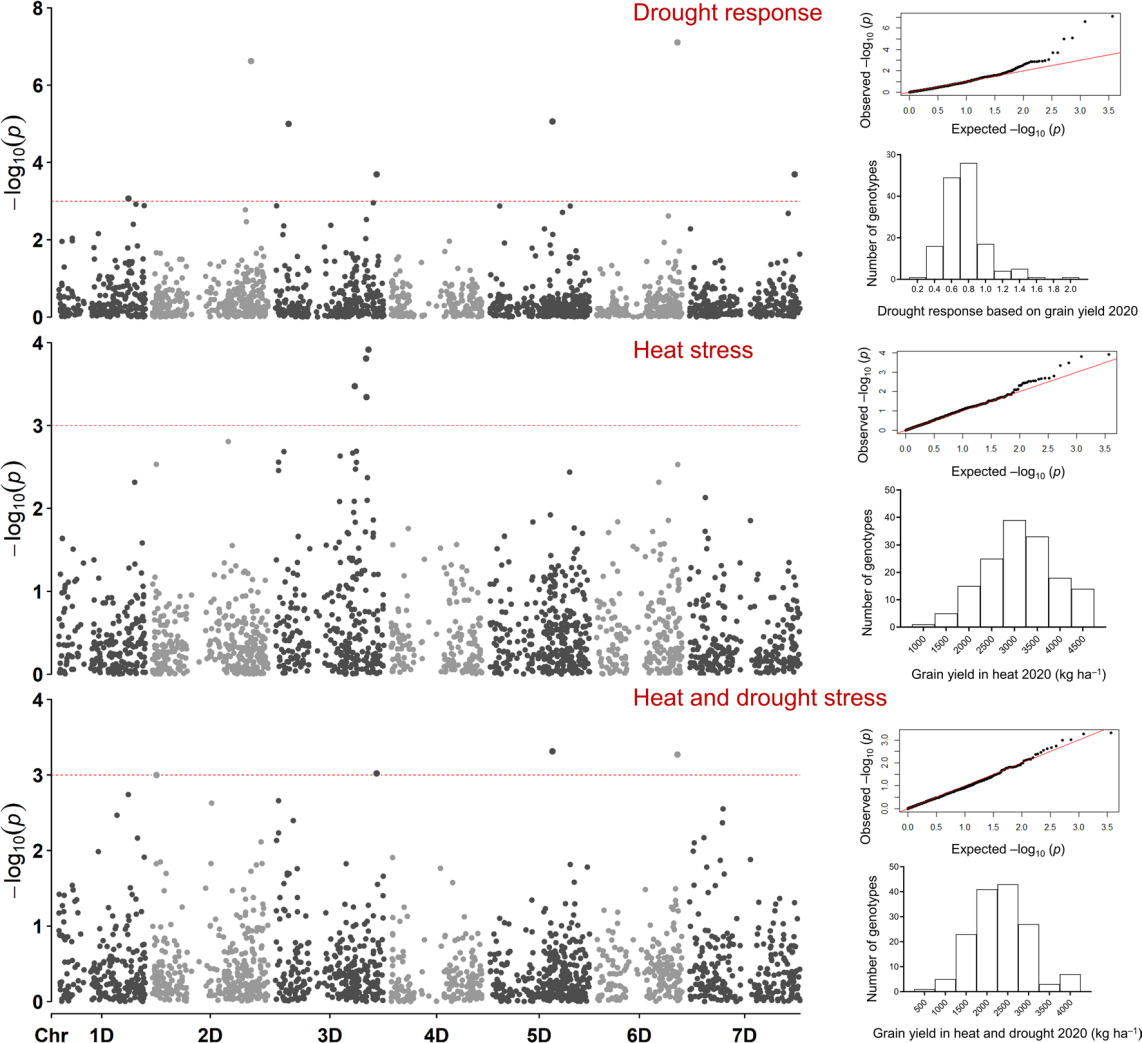
Representative Manhattan plots for grain yield showing marker–trait associations in the D genome of bread wheat lines under heat or combined heat–drought stress, and in the drought response. The distribution of grain yield and quantile–quantile plots of the genome-wide analysis are shown for each condition

**Fig. 5:**
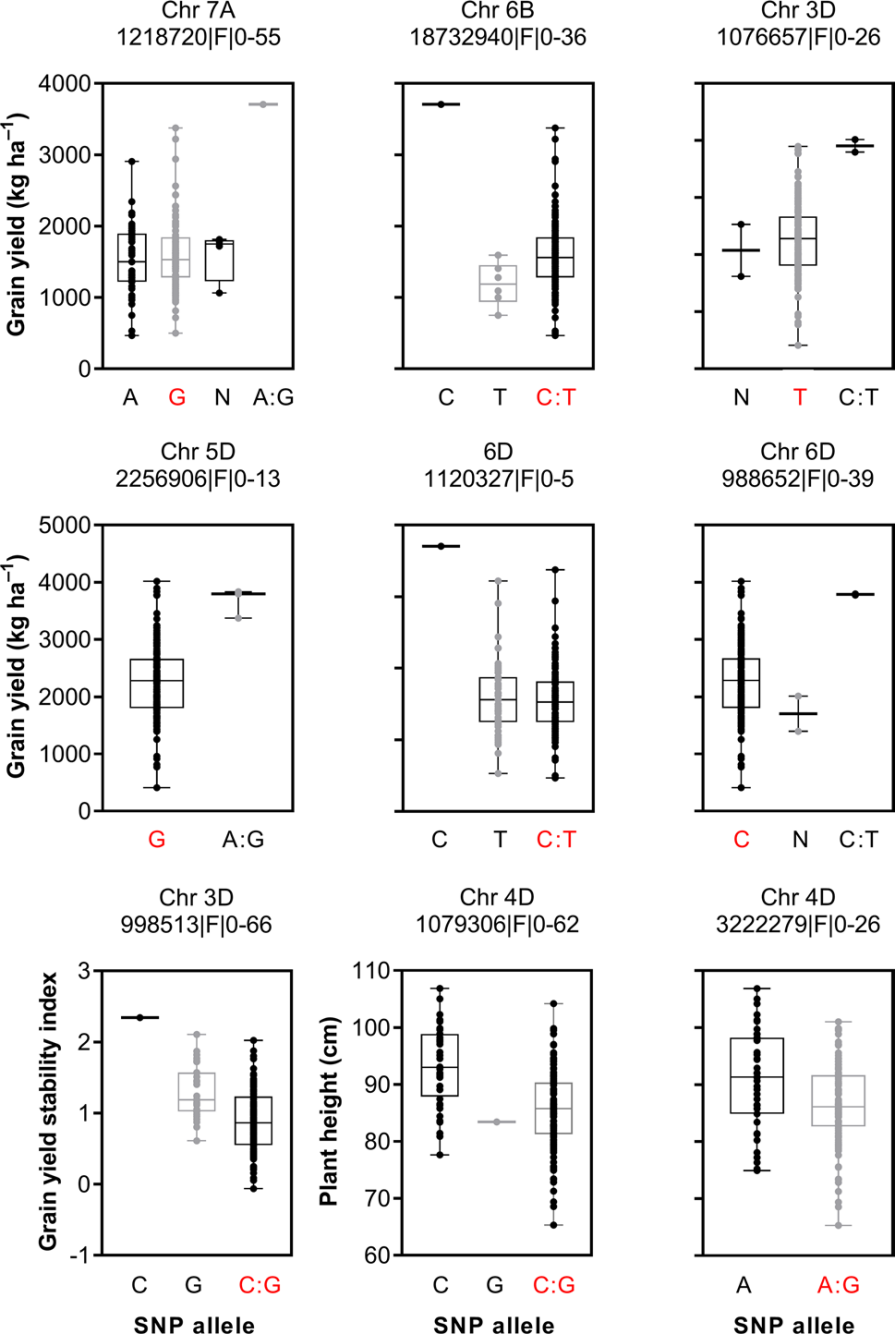
Effect of selected stable marker–trait associations on grain yield, grain yield stability index, and plant height in a bread wheat population grown under heat or combined heat–drought stress. A, adenine; C, cytosine; T, thymine; G, guanine; N, unknown. Red alleles are those of the backcross parent of the population, ‘Norin 61’

Since the same MTAs were obtained from both adjusted and unadjusted BLUEs, only the MTAs from the adjusted BLUEs are presented. Also, since all the MTAs found in the whole-genome GWAS were again found in the D genome-only GWAS, the MTAs with lowest FDR from either GWAS were selected (Table S5). A sample of results of the GLM and MLM models for both GWAS under HD in 2020 are shown in Table S6. Many significant MTAs were identified for various traits, with 12% and 27% of the MTAs passing the FDR 0.05 and 0.2 thresholds, respectively. In 2019, 75 MTAs for H, 4 for HD, and 25 for DR passed the FDR tests; in 2020, 23 MTAs for H, 62 for HD, and 123 for DR passed (Fig. S1a). In total, 100 highly significant MTAs (FDR < 0.2) were associated with H, 68 with HD, and 150 with DR in both years (including MTAs for GY stability index, Table S5). A summary of the MTAs (except those for GC, SPAD, and δ^13^C) and chromosomal positions is shown in Fig. 3. The MTAs were found on 19 chromosomes, and 56.6% of all MTAs were found on chromosomes (Chrs.) 2D, 3D, 5D, and 7D (Fig. S1b). About 70.0% of all MTAs were for GY, KPS, NDVI, and CT (Fig. S1c). The MTAs identified for CT1, CT3, GY, KPS, and PH had the highest variation in allelic effect, which ranged from 6.7% to 52.1% (Fig. S1d). All identified MTAs are listed in Table S5. Representative Manhattan plots are shown in Fig. S2, and those for GY in the D genome alone are shown in Fig. 4. We identified 41 stable or pleiotropic MTAs (Table 2). Further analysis of some of the stable MTAs revealed the source of the SNP alleles (*Ae. tauschii*, ‘Langdon’, or N61) and the possible effect of the alleles on individual traits (Fig. 5). The positive alleles for PH, GY stability, and GY on the D genome originated from *Ae. tauschii* (Fig. 5).

**Table 2:** Stable and pleiotropic MTAs under heat (H) and combined heat–drought (HD) conditions, and in the drought response (DR) in 2019 and 2020

Environment	Year	FDR threshold	Trait (specific environment)	Marker	Chr	Position (Mbp)	Allelic effect (%)
DR	2019	0.2	GY, HI	3,937,862|F|0–24	1D	58.724191	12.8–13.7
HD	2020	0.2	GY, HI	3,020,847|F|0–49	2D	23.110173	7.6–8.5
H, HD, DR	2020	0.05	GY, SI, NDVI	998,513|F|0–66	3D	508.32788	11.2–20.3
HD, DR	2020	0.05	GY	1,076,657|F|0–26	3D	567.58126	7.7–9.7
HD, DR	2020	0.05	GY	2,256,906|F|0–13	5D	356.61467	8.5–13.5
H, DR	2019, 2020	0.2	GY (DR2019), GFD(H2020)	1,120,327|F|0–5	6D	295.33974	11.4–15.5
HD, DR	2020	0.05	GY	988,652|F|0–39	6D	452.87905	8.5–19.3
H, HD	2019	0.2	GY	1,218,720|F|0–55	7A	557.24618	14.6–22.1
HD, DR	2019	0.2	GY, BIO (HD2019)	18,732,940|F|0–36	6B	69.312667	12.9–22.1
HD	2020	0.05	TKW, NDVI	1,161,247|F|0–23	2D	614.01796	7.7–12.3
HD	2020	0.2	TKW, NDVI	4,734,029|F|0–28	2D	614.17744	7.1–9.2
H, DR	2019, 2020	0.05	TKW (H2019), KPS (DR2020)	1,057,222|F|0–51	3D	90.289806	9.3–18.9
HD, DR	2019, 2020	0.2	TKW (HD2020), KPS (DR2020), CT1(HD2019)	985,748|F|0–43	3D	550.71378	9.1–15.3
DR	2020	0.05	TKW, KPS	7,351,923|F|0–56	5D	240.6867	7.4–9.6
DR	2020	0.05	TKW, KPS	1,230,357|F|0–39	5D	240.6867	7.3–9.6
HD	2020	0.05	TKW, NDVI	1,385,391|F|0–63	2D	606.42916	10.9–15.5
HD	2020	0.2	TKW, NDVI	1,228,058|F|0–36	2D	607.16567	9.7–10.5
HD	2020	0.05	TKW, NDVI	1,161,247|F|0–23	2D	614.01796	7.7–12.3
HD	2020	0.2	TKW, NDVI	4,734,029|F|0–28	2D	614.17744	7.1–9.2
HD	2020	0.05	TKW, NDVI	1,385,391|F|0–63	2D	606.42916	10.8–15.521
H, HD	2020	0.2	KPS, CT1, NDVI	994,213|F|0–21	2D	511.70796	11.2–13.3
DR	2020	0.2	BIO, CT2	991,074|F|0–56	3D	43.382158	8.9–12.6
HD, DR	2020	0.2	BIO (DR), CT2	986,326|F|0–60	5D	359.1114	9.3–10.8
HD, DR	2020	0.05	BIO (DR), CT2	3,028,230|F|0–33	5D	413.71439	7.7–10.7
HD, DR	2020	0.2	BIO (DR), CT2	1,012,073|F|0–63	7D	556.34431	8.9–10.7
H, HD, DR	2019, 2020	0.05	PH	1,079,306|F|0–62	4D	25.701834	15.2–27.3
H, HD	2020	0.2	PH	4,005,784|F|0–33	4D	36.86672	7.2–9.7
DR	2020	0.2	DM, GFD	991,772|F|0–64	3D	507.14502	7.9–8.0
HD, DR	2020	0.05	CT2	2,251,455|F|0–29	3D	55.135592	12.6–14.7
HD, DR	2020	0.05	CT3	12,002,285|F|0–11	1D	315.11177	10.6–12.3
H, HD	2020	0.2	CT3, NDVI	1,015,501|F|0–10	3D	164.82545	10.2–12.6
H, DR	2019, 2020	0.2	CT3, NDVI	2,250,763|F|0–61	5D	410.7371	9.0–11.8
HD, DR	2020	0.05	SPAD	1,236,663|F|0–12	5D	344.28537	16.8–17.3
HD	2020	0.2	NDVI	1,228,058|F|0–36	2D	607.16567	9.7–10.5

Traits without an environment in parenthesis were identified in the environment(s) and year(s) listed in the first and second columns, respectively. BIO, biomass; CT1, canopy temperature 7 days before flowering; CT2, canopy temperature at flowering; CT3, canopy temperature at grain filling; DM, days to maturity; GFD, grain-filling duration; GY, grain yield; HI, harvest index; KPS, kernel number per spike; NDVI, normalized difference vegetation index; PH, plant height; SI, GY stability index; SPAD, chlorophyll content; TKW, thousand-kernel weight

### MTAs under H and HD

Under H, 88.5% of the MTAs identified were for CT1, CT3, PH, NDVI, or GFD. Ten MTAs for CT3 explained between 19.8% and 35.1% of the variation, and six of them were collocated between 710 and 718 Mbp on Chr. 3A (Table S5). Two MTAs for GY (7,940,688|F|0–16 and 998,513|F|0–66), located between 508 and 522 Mbp on Chr. 3D, explained 10.6% and 11.8%, respectively, of the phenotypic variation (Fig. 3, Table S5).

Under HD, 55% of the MTAs identified were for CT1, NDVI, PH, and KPS. Nine MTAs on Chrs. 1D, 3D, 5D, and 7D were identified for CT1, seven of which were on 3D and 7D and explained 7.4%–11.4% of the variation (Fig. 3, Table S5). Among nine MTAs for NDVI, six were collocated on Chr. 2D (606–618 Mbp) and explained 9.2%–15.4% of the variation. Twelve MTAs were identified for KPS, three of which were collocated on Chr. 7D (345–384 Mbp) and explained on average 9.9% of the variation. Six MTAs for GY explained 7.8%–14.5% of the variation (Fig. 3, Table S5).

### MTAs for DR

To estimate DR, the ratio of BLUEs obtained under HD to those obtained under H was used for GWAS. Although this ratio tends to oversimplify the complex relationship between heat and drought stress, it increased the statistical power of the analysis enabling the detection of many potentially important MTAs. These MTAs explained, on average, 14.7% of the phenotypic variation and 51.8% of the MTAs controlled GY and KPS. The MTAs for GY explained 7.2%–24.1% of the variation, whereas MTAs for KPS explained 7.10%–40.4%. Six MTAs for KPS were collocated: 7,351,923|F|0–56 and 1,230,357|F|0–39 were located at 240.6 Mbp on Chr. 3B, 1,056,569|F|0–52 and 981,730|F|0–67 at 333.1 Mbp on Chr. 5D, and 2,252,899|F|0–22 and 3,024,415|F|0–22 at 408.0–408.6 Mbp on Chr. 5D (Fig. 3, Table S5). Two MTAs for GY (1,125,420|F|0–29 and 1,072,095|F|0–54) were collocated on Chr. 3D and explained 21.5% and 9.6%, respectively, of the variation.

### Loci controlling plant phenology

MTAs for PH were identified on Chrs. 4A and 3D–7D. Two MTAs (1,001,495|F|0–20 and 1,042,486|F|0–52) were collocated between 577.5 and 577.7 Mbp on Chr. 4A and explained 7.2%–9.7% of the variation under H and HD conditions. Four MTAs controlling PH were found between 25 and 37 Mbp on Chr. 4D. Two of them were stable across H and HD: 1,079,306|F|0–62 was stable in both years and explained 9.5–25.7% of the variation, whereas 4,005,784|F|0–33 was stable in 2020 and explained 7.2–9.7% of the variation in PH (Table S5). Eleven MTAs were identified for GFD and explained, on average, 10.3% of the variation. An MTA on Chr. 3D (991,772|F|0–64) was pleiotropic for DM in the DR in 2020, and an MTA on Chr. 6D (1,120,327|F|0–5) was pleiotropic for GY under H in 2020 (Table 2).

### Loci controlling leaf traits associated with GY traits

Because leaf traits were correlated with GY and related traits (Table S3), we investigated the loci controlling CT, NDVI, and δ^13^C. The heritability estimates for CT measurements were low, even zero in some cases, while those for δ^13^C could not be determined since δ^13^C was measured in only one season (Table 1). However, due to the importance of these traits for wheat breeding in hot, dry areas, we conducted GWAS analysis and found potentially useful MTAs. Most of the MTAs for leaf traits were found on Chrs. 2D, 3D, and 5D. Three loci were identified on Chr. 2D: the first locus (23–96 Mbp) harbored MTAs for δ^13^C, CT3, NDVI, and HI, which explained 7.6%–30.6% of the variation; the second locus (511–554 Mbp) harbored MTAs for CT1, NDVI, KPS, and GY, which explained 7.6–17.7%; and the third locus (606–614 Mbp) harbored MTAs for NDVI, TKW, and GY, which explained 7.1%–16.2%. The locus on Chr. 3D (155–171 Mbp) harbored MTAs for CT1, CT3, and NDVI, which explained on average 9.9% of the variation. The locus on Chr. 5D (407–413 Mbp) contained MTAs for CT1, CT2, CT3, NDVI, and BIO, which explained 7.7%–32.8% of the variation. As all these loci on Chrs. 2D, 3D, and 5D also harbored MTAs for GY and related traits under H, HD, and DR (Fig. 3, Table S5), they may be important for yield improvement for H and drought stress resilience.

### Loci controlling GY, KPS, and TKW

We identified a locus on Chr. 3D (521–549 Mbp) controlling GY and explaining 9.6%–21.5% of the variation under H and DR, and another locus on Chr. 3D (79–90 Mbp) common to KPS and TKW explaining 9.3–22.4% of the variation under H and DR (Table S5). We identified another locus common to KPS and TKW on Chr. 5D (240.6 Mbp) explaining on average 8.5% of the variation in DR, and a locus controlling GY and KPS on Chr. 7A (517–556 Mbp) explaining a relatively high (21.0%–40.3%) proportion of the variation under DR.

### Candidate genes

The markers in the AB genome are in high LD with LD decay extending up to 131 Mbp, whereas those in the D genome have an LD decay at 1.1 Mbp. A candidate gene for GY, GY stability, and NDVI was identified as a gibberellin-regulating gene (*GA20ox* TraesCS3D02G393900) on Chr. 3D (998,513|F|0–66, 508.3 Mbp). A candidate gene for CT3 was identified as a gene involved in CaaX prenylation (*CaaX prenyl protease 2*, TraesCS1D02G228400) on Chr. 1D (12,002,285|F|0–11, 315.1 Mbp). Their expression patterns show highest expression in the endosperm (*GA20ox,* Fig. S3) and in the roots, shoots and inflorescences (*CaaX prenyl protease 2*, Fig. S4). These relative expressions were sourced from many independent studies involving different genotypes, growth stages, and conditions implemented in Genevestigator. A list of the candidate genes is shown in Table S7.

## Discussion

The strong G × E interaction effect (*p* < 0.001, Table S2) on most traits confirmed the high genetic diversity of the panel. Under HD, GY was drastically reduced (up to 58% of that under H), highlighting the detrimental effect of HD under natural field conditions. Similar observations have been reported under field (Pradhan et al. 2012; Liu et al. 2019) and controlled conditions (Prasad et al. 2011; Schmidt et al. 2020). The moderate to high heritability estimates for most of the traits were similar to those in Sukumaran et al. (2018) under separate H, drought stresses, and yield potential conditions in Mexico, reflecting the genetic control of these traits across environments. The correlations observed between most of the evaluated traits suggest an association among these traits at the genetic level, which improves selection efficiency (Shimelis and Shiringani 2010). The weak positive correlations of individual traits between H and HD indicate that genotypes that performed well under H were strongly affected by HD (Table S3). However, some genotypes performed relatively well under both conditions and are promising for further breeding (Fig. 2). Their good performance may be attributed to the effect of different SNP alleles. For example, genotypes with the R allele at 1,218,720|F|0–55 on Chr. 7A, C allele for 18,732,940|F|0–36 on Chr. 6B, and Y allele for 1,076,657|F|0–26 on Chr. 3D had higher GY across the environments than that of other genotypes, including N61 (Fig. 5).

Among the leaf traits, CT1–CT3 and δ^13^C showed negative correlation trends with GY and related traits, whereas NDVI showed positive correlation trends, especially with BIO (Table S3). Similar results have been reported under field conditions (Rutkoski et al. 2016; Sukumaran et al. 2018), indicating the effectiveness of these traits for improving selection efficiency for GY. Rutkoski et al. (2016) reported the use of CT and NDVI measurements to improve GY prediction accuracy by up to 70%, whereas an earlier study in Spain (Royo et al. 2002) reported that carbon isotope discrimination was more effective than canopy temperature depression in assessing genotypic variation in GY. Itam et al. (2020b) reported an increasing trend of CT and δ^13^C under progressive drought stress due to stomatal regulation. Overall, a combination of CT, NDVI, and δ^13^C will further improve the accuracy of selection for GY in wheat.

The genetic basis of quantitative traits such as H and HD resilience is complex and requires detailed genomic analyses. In GWAS, we set DH as a covariate to minimize any possible confounding effect of plant phenology. Many MTAs are reportedly dependent on DH (Sukumaran et al. 2018; Schmidt et al. 2020), but 99% of the MTAs identified here were independent of DH, mainly because the selection of our wheat panel was based on similar DH. No significant correlation was found between DH and GY (Table S3). Therefore, we consider this wheat panel to be suitable for mining novel QTLs for H and HD stress resilience without the confounding effect of plant phenology. We found stable MTAs across two or more conditions and pleiotropic MTAs controlling more than one trait, indicating the stability of the associated QTLs across environments and common regulation of these traits (Tables 2, S5). A plot of stable MTAs showed the effect of SNP alleles on the evaluated traits, indicating that positive alleles for GY on Chrs. 6B and 7A were derived from ‘Langdon’, the durum wheat cultivar used as a bridge during the crosses (Fig. 5). Itam et al. (2021) reported that the introgressed segments from ‘Langdon’ (AABB) contribute to the A and B genome diversity of the panel. Conversely, the positive alleles in the D genome derived from *Ae. tauschii* were associated with high GY stability index and GY under H and HD (Fig. 5). Some MSD lines and N61 contained a negative allele for PH, supporting the fact that N61 harbors the dwarfing genes that were important for the Green Revolution (Tsujimoto 2021). Overall, genotypes carrying the positive alleles for GY and GY stability index and negative alleles for PH may be selected for future breeding.

### Genetic control of leaf traits

QTLs on Chrs. 2D (23–47, 511–554, and 606–614 Mbp), 3D (155–171 Mbp), and 5D (407–413 Mbp) were identified for most leaf traits, including CT1–CT3 and NDVI. The Chr. 2D locus at 606–614 Mbp (130.8 cM) was previously reported for GY under HD in a synthetic-derived parent/elite line RIL population (Liu et al. 2019) and is potentially novel for NDVI. The Chr. 2D locus at 23–47, (20.9 cM) was previously reported for CT depression in recombinant inbred lines under heat stress (Mondal et al. 2015), for GY in doubled haploids under heat stress (Bennett et al. 2012), and for carbon isotope discrimination in doubled haploids under rainfed and irrigated environments (Rebetzke et al. 2008). In this study, the Chr. 2D locus at 96 Mbp controlled the δ^13^C value, an important physiological trait for evaluating stress response. However, owing to the limitations of δ^13^C or carbon isotope discrimination (Dixon et al. 2019), direct selection using carbon isotope traits alone may offer limited opportunities for wheat improvement. In this study, heritability estimates for CT were low, while those for δ^13^C could not be determined (Table 1). Therefore, the QTLs for CT and δ^13^C only show a potential trend and must be carefully validated before utilization. Taken together, a combination of δ^13^C and the easy-to-measure leaf traits such as CT and NDVI will likely improve selection efficiency.

One candidate gene regulating canopy temperature at grain filling (CT3) was *CaaX prenyl protease 2* (TraesCS1D02G228400) (Fig. S4). CaaX prenyl proteases are involved in the prenylation of CaaX proteins, a step essential for protein–membrane interactions, plant development, and stress signaling, especially in abscisic acid signaling in Arabidopsis (Bracha-Drori et al. 2008) and wheat (Zhang et al. 2015). In the bread wheat lines, this gene may play a role in stress signaling and stomatal regulation under H and HD stresses, resulting in canopy temperature regulation. Further research is needed for the applicability of this gene to wheat breeding. QTLs for CT promote downward root growth (30–90 cm) under drought stress and root spread close to the soil surface under H, a root distribution strategy for wheat adaptation to both stresses (Pinto and Reynolds 2015). However, HD would likely result in a tradeoff between root elongation and spread to optimize plant–water relations. In our study, the decreasing trend of CT2 and CT3 suggests that most of the wheat lines maintained lower canopy temperature under HD than in H stress. Low canopy temperature has been linked to high GY in wheat under separate H and drought stress conditions (Pinto and Reynolds 2015). The QTLs on Chrs. 2D, 3D, and 5D may regulate resilience to H and HD and are potentially useful for wheat breeding.

### Genetic control of GY and related traits

In wheat, GY is the most important trait. A locus on Chr. 3D (521–549 Mbp) controlled GY alone, a locus on Chr. 7A (517–556 Mbp) controlled GY and KPS, and two loci on Chrs. 3D (79–90 Mbp) and 5D (240.6 Mbp) each controlled KPS and TKW under H and HD stresses. The Chr. 7A loci (517–556 Mbp, 80.1–83.3 cM) was previously reported for GY in elite European varieties under moderate water deficit (Touzy et al. 2019). Information on other loci is not clear. Taken together, these loci explained up to 40.3% of the variation in GY and related traits under H and HD and thus they offer great potential for improvement of wheat climate resilience. This potential reflects the importance of harnessing *Ae. tauschii* diversity for climate resilience breeding using the synthetic derivative approach (Tsujimoto et al. 2015). Further analysis and validation of individual QTLs using a recombinant population would be needed to better understand the effects of the pleiotropic QTLs on individual traits. Also, the use of functional markers such as kompetitive allele-specific PCR (Fang et al. 2020; Rasheed et al. 2016) markers may facilitate selection and further breeding.

It is worthy of note that an MTA for GY stability index on Chr. 3D (998,513|F|0–66, 508.3 Mbp) was linked to the gene TraesCS3D02G393900, which is orthologous to *gibberellin-20-oxidase* (*GA20ox*) in *Zea may*s L. (Zm00001d007894). *GA20ox3* functions in gibberellin biosynthesis, and gibberellins play a central role in plant responses to abiotic stresses by integrating multiple hormone signaling pathways (Colebrook et al. 2014). Similarly, gibberellin-sensitive *Rht* alleles (controlling plant height) have been reported to confer tolerance to heat and drought stress in wheat (Alghabari et al. 2016). We hypothesize that TraesCS3D02G393900 on Chr. 3D may favorably alter gibberellin content, ultimately resulting in higher GY stability under H and HD conditions. This offers a potential for developing climate-resilient wheat cultivars by optimizing gibberellin homeostasis. However, further investigations are needed to test this hypothesis by exploiting the diversity in gibberellin-regulating genes in wheat. Our database search revealed that TraesCS3D02G393900 is mainly expressed during the late vegetative and reproductive stages, with highest expression in the endosperm (Fig. S3) (Pearce et al. 2015). Similar expression patterns were reported in its orthologs in *Z. mays* (Zm00001d007894, Yousaf et al. 2019), *Oryza sativa* L. (LOC_Os07g07420, Qin et al. 2013), and *Hordeum vulgare* L. (HORVU3Hr1G089980, Betts et al. 2020) indicating a similar function of these genes among members of the grass family. Moreover, as the candidate genes were found at a distance less than the LD decay (LD decay for D genome = 1.1 Mbp, for AB genome = 131 Mbp), they may be useful for further breeding.

## Conclusion

The wheat MSD panel used in this study represents the diversity of 37 *Ae. tauschii* accessions, and the study provides insights into the utilization of high-diversity breeding panels for wheat improvement. Since GWAS studies under HD in field conditions are scarce, the identified candidate genes, alleles, and QTLs will potentially serve as genomic landmarks for breeding to improve wheat adaptation to H and HD stresses under climate change.

## Supplementary Information

Below is the link to the electronic supplementary material.

Summary of marker–trait associations (MTAs). (a) Number of MTAs in each field condition in 2019 and 2020; (b) number of MTAs identified on each chromosome; (c) number of MTAs identified for each trait; and (d) the range of allelic effects of MTAs in each trait. BIO, biomass; CT1, canopy temperature at 7 days before flowering; CT2, canopy temperature at flowering; CT3, canopy temperature at grain filling; DH, days to 50% heading; DM, days to maturity; GC, ground cover; GFD, grain-filling duration; GY, grain yield; HI, harvest index; KPS, kernel number per spike; NDVI, normalized difference vegetation index; PH, plant height; SI, GY stability index; SN, number of spikes per plot; SPAD, chlorophyll content; TKW, thousand-kernel weight; δ13C, delta carbon-13 value (TIF 2273 kb)

Representative Manhattan plots of plant height (PH) and grain yield (GY) showing marker–trait associations in all three subgenomes of bread wheat lines under combined heat and drought stress (for PH) and in the drought response (for GY). The quantile–quantile plots of the genome-wide analysis are shown on the right (TIF 3239 kb)

Expression pattern of GA20ox (TraesCS3D02G393900), the candidate gene for grain yield stability in wheat. The expression in 55 anatomical parts is shown on a log2 scale and is relatively high in endosperm tissues. Data were retrieved from the wheat transcriptome database (mRNA-Seq Gene Level Triticum aestivum, ref: IWGSCv1.1) implemented in Genevestigator. The data contains independent studies involving different genotypes, growth stages, and conditions (PDF 30 kb)

Expression pattern of CaaX prenyl protease 2 (TraesCS1D02G228400), the candidate gene for canopy temperature at grain filling in wheat. The expression in 55 anatomical parts is shown on a log2 scale and the is relatively high in roots, shoots, and inflorescences. Data were retrieved from the wheat transcriptome database (mRNA-Seq Gene Level Triticum aestivum, ref: IWGSCv1.1) implemented in Genevestigator. The data contains independent studies involving different genotypes, growth stages, and conditions (PDF 37 kb)

List of plant materials. The pedigrees of the MSD lines include the origins and lineages of their donor *Ae. tauschii* accessions (XLSX 16 kb)

Mean sums of squares for traits evaluated under heat and combined heat–drought stress in 2019 and 2020 (XLSX 12 kb)

Correlation coefficients of the evaluated traits in 2019 and 2020 (XLSX 20 kb)

MSD lines selected on the basis of grain yield (GY) and its stability under heat (H) and combined heat–drought (HD) stress in comparison with their backcross parent (Norin 61) and a Sudanese check cultivar (Imam) (XLSX 12 kb)

Marker–trait associations detected under heat (H) and combined heat–drought (HD) conditions, and in the drought response (DR) in 2019 and 2020 (XLSX 31 kb)

A sample of results of the GLM and MLM models for general and D-genome GWAS under the combined heat–drought (HD) conditions for 2020 (XLSX 43 kb)

Selected candidate genes for marker–trait associations and their locations and functions (XLSX 12 kb)

## Declarations

### Availability of data and material

The genomic data are available at the Dryland Public Research Database of Tottori University (https://docs.google.com/spreadsheets/d/1tmihGw-xZqKt7NUtCqca-_vzVIavvJXm/edit?usp=sharing&ouid=112779701721431756615&rtpof=true&sd=true).
