# Supplementary figures and images for: Genomic analysis for heat and combined heat–drought resilience in bread wheat under field conditions
Source: Theor Appl Genet. 2021 Oct 16;135(1):337–50. doi: 10.1007/s00122-021-03969-x (PMC8741676; doi:10.1007/s00122-021-03969-x)

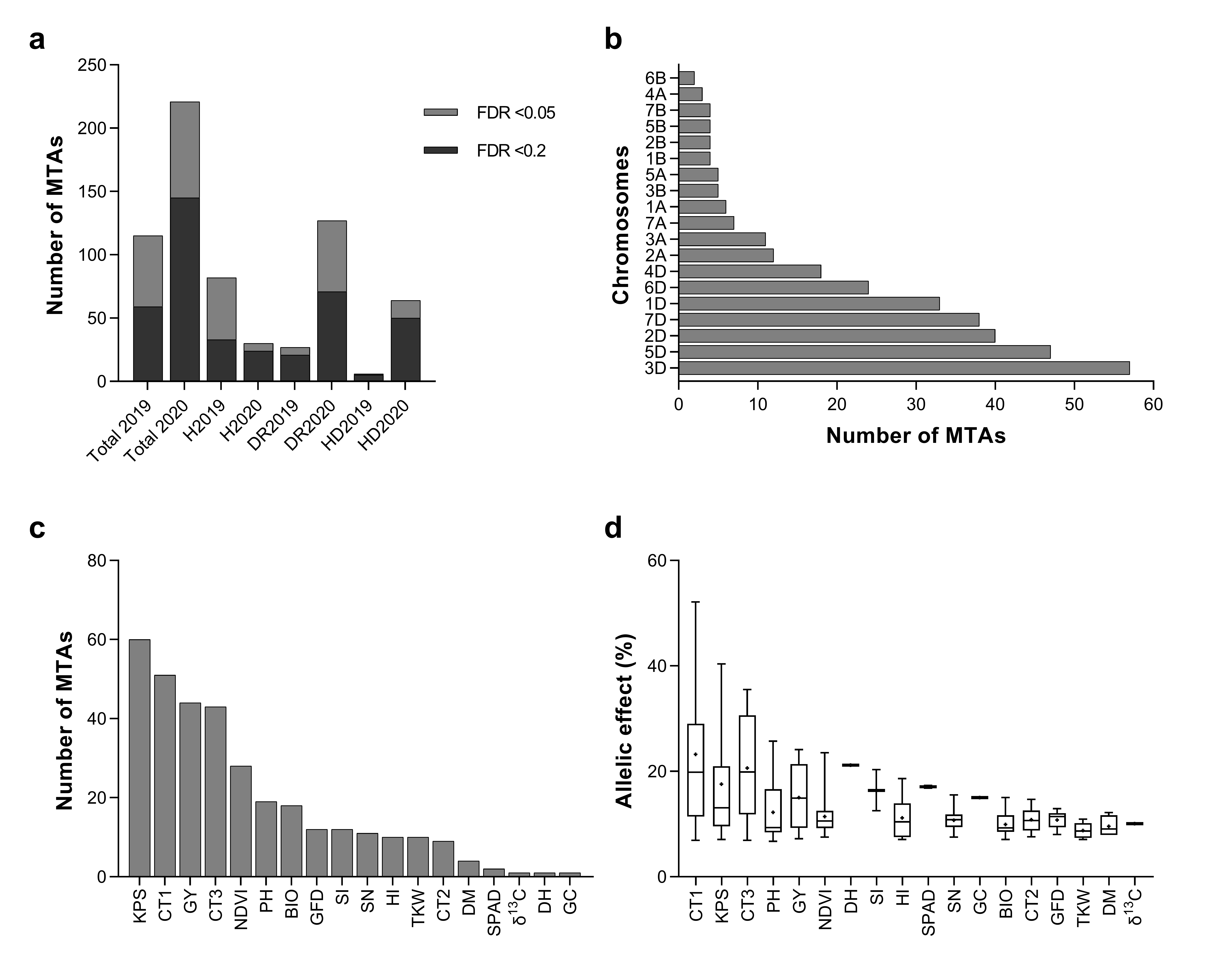

Supplement: Supplementary file 1 — Summary of marker–trait associations (MTAs). (a) Number of MTAs in each field condition in 2019 and 2020; (b) number of MTAs identified on each chromosome; (c) number of MTAs identified for each trait; and (d) the range of allelic effects of MTAs in each trait. BIO, biomass; CT1, canopy temperature at 7 days before flowering; CT2, canopy temperature at flowering; CT3, canopy temperature at grain filling; DH, days to 50% heading; DM, days to maturity; GC, ground cover; GFD, grain-filling duration; GY, grain yield; HI, harvest index; KPS, kernel number per spike; NDVI, normalized difference vegetation index; PH, plant height; SI, GY stability index; SN, number of spikes per plot; SPAD, chlorophyll content; TKW, thousand-kernel weight; δ13C, delta carbon-13 value (TIF 2273 kb) [file 122_2021_3969_MOESM1_ESM.tif]

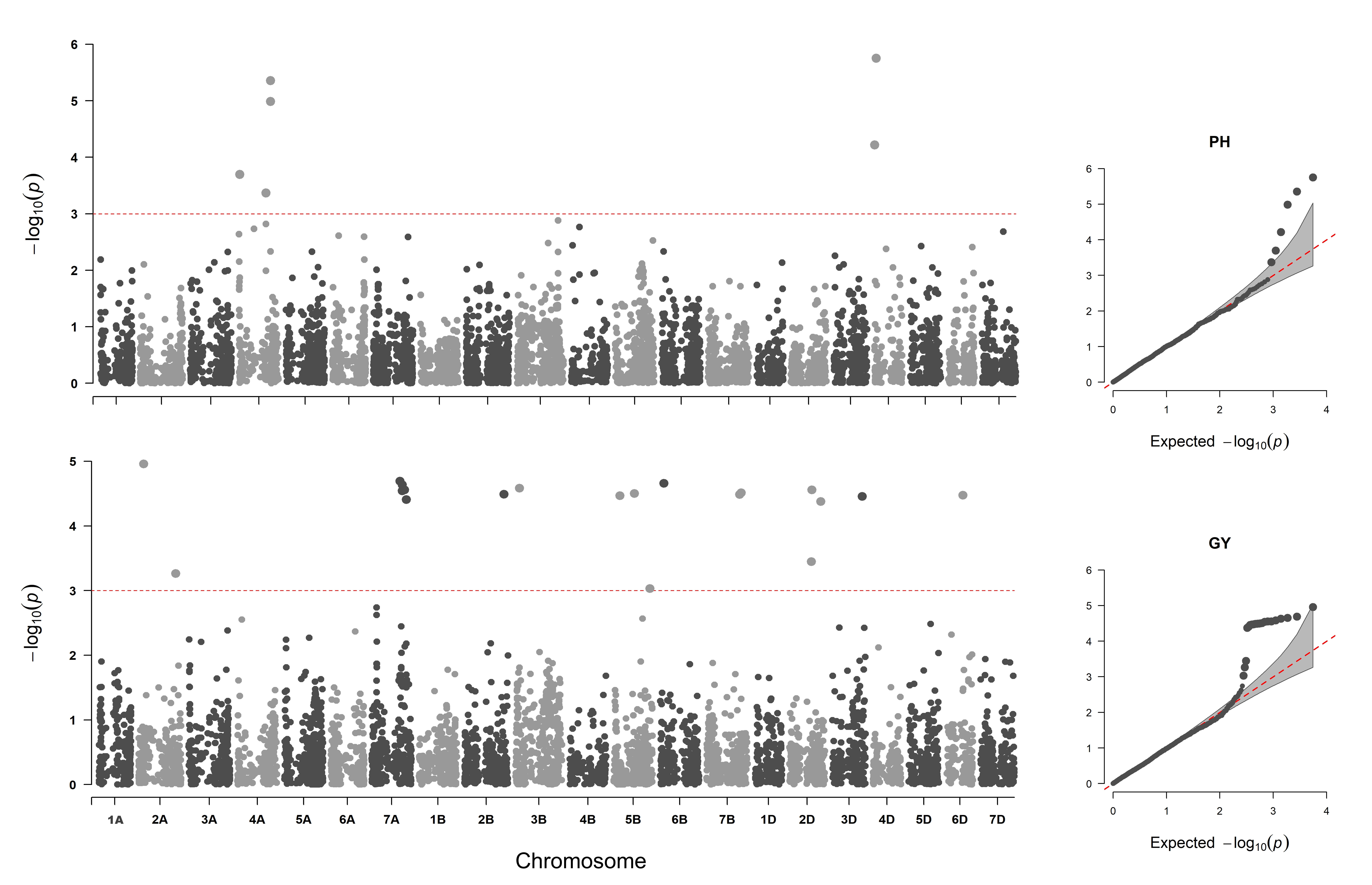

Supplement: Supplementary file 2 — Representative Manhattan plots of plant height (PH) and grain yield (GY) showing marker–trait associations in all three subgenomes of bread wheat lines under combined heat and drought stress (for PH) and in the drought response (for GY). The quantile–quantile plots of the genome-wide analysis are shown on the right (TIF 3239 kb) [file 122_2021_3969_MOESM2_ESM.tif]
