# Supplementary material for: Genomic analysis for heat and combined heat–drought resilience in bread wheat under field conditions
Source: Theor Appl Genet. 2021 Oct 16;135(1):337–50. doi: 10.1007/s00122-021-03969-x (PMC8741676; doi:10.1007/s00122-021-03969-x)
Supplement: Supplementary file 3 — Expression pattern of GA20ox (TraesCS3D02G393900), the candidate gene for grain yield stability in wheat. The expression in 55 anatomical parts is shown on a log2 scale and is relatively high in endosperm tissues. Data were retrieved from the wheat transcriptome database (mRNA-Seq Gene Level Triticum aestivum, ref: IWGSCv1.1) implemented in Genevestigator. The data contains independent studies involving different genotypes, growth stages, and conditions (PDF 30 kb) [file 122_2021_3969_MOESM3_ESM.pdf]

Dataset: 55 anatomical parts from data selection: TA_mRNASeq_WHEAT_GL-0

Showing 1 measure(s) of 1 gene(s) on selection: *GA20ox*

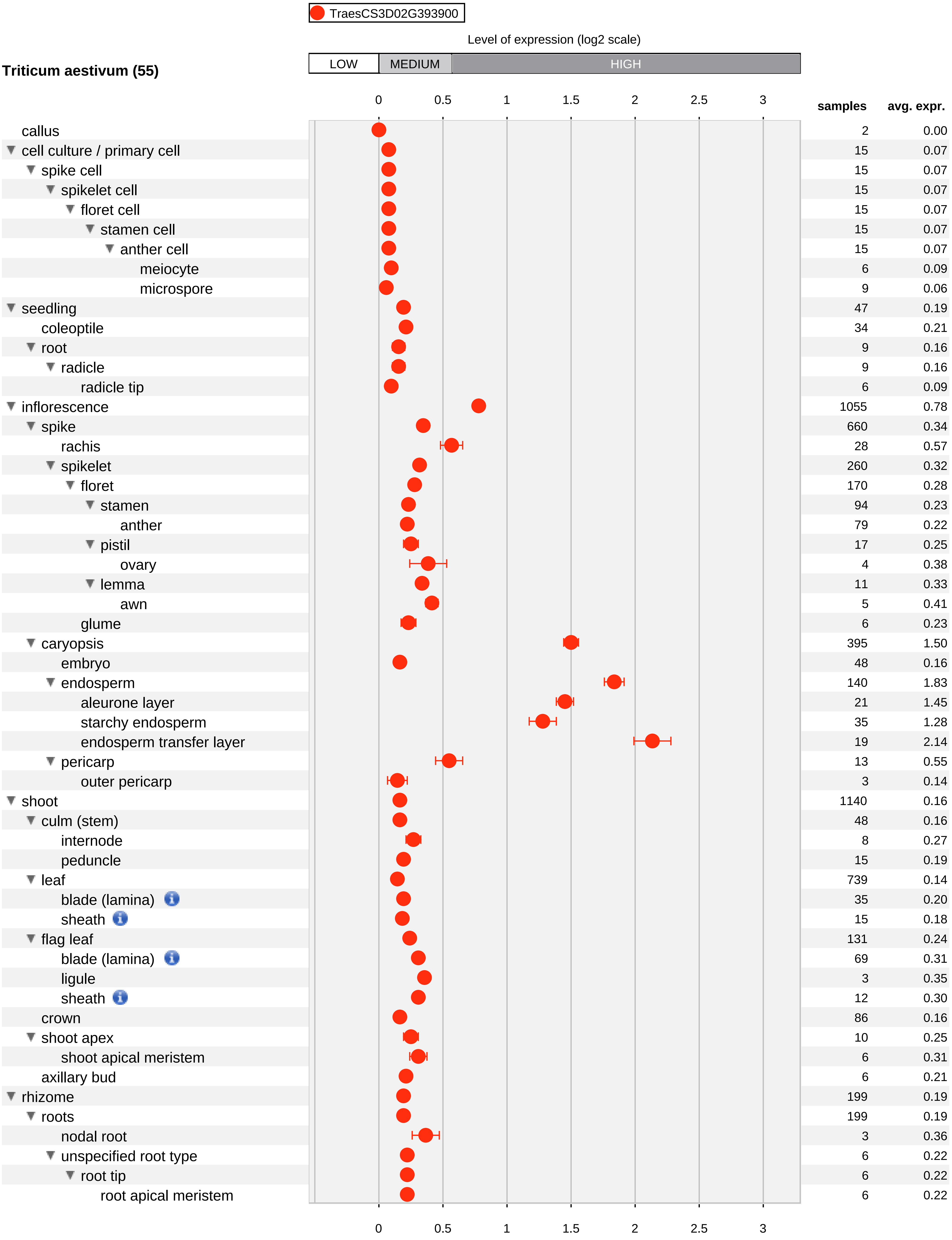

created with GENEVESTIGATOR
