# Supplementary material for: Genomic analysis for heat and combined heat–drought resilience in bread wheat under field conditions
Source: Theor Appl Genet. 2021 Oct 16;135(1):337–50. doi: 10.1007/s00122-021-03969-x (PMC8741676; doi:10.1007/s00122-021-03969-x)
Supplement: Supplementary file 4 — Expression pattern of CaaX prenyl protease 2 (TraesCS1D02G228400), the candidate gene for canopy temperature at grain filling in wheat. The expression in 55 anatomical parts is shown on a log2 scale and the is relatively high in roots, shoots, and inflorescences. Data were retrieved from the wheat transcriptome database (mRNA-Seq Gene Level Triticum aestivum, ref: IWGSCv1.1) implemented in Genevestigator. The data contains independent studies involving different genotypes, growth stages, and conditions (PDF 37 kb) [file 122_2021_3969_MOESM4_ESM.pdf]

Dataset: 55 anatomical parts from data selection: TA_mRNASeq_WHEAT_GL-0

Showing 1 measure(s) of 1 gene(s) on selection: CaaX prenyl protease2

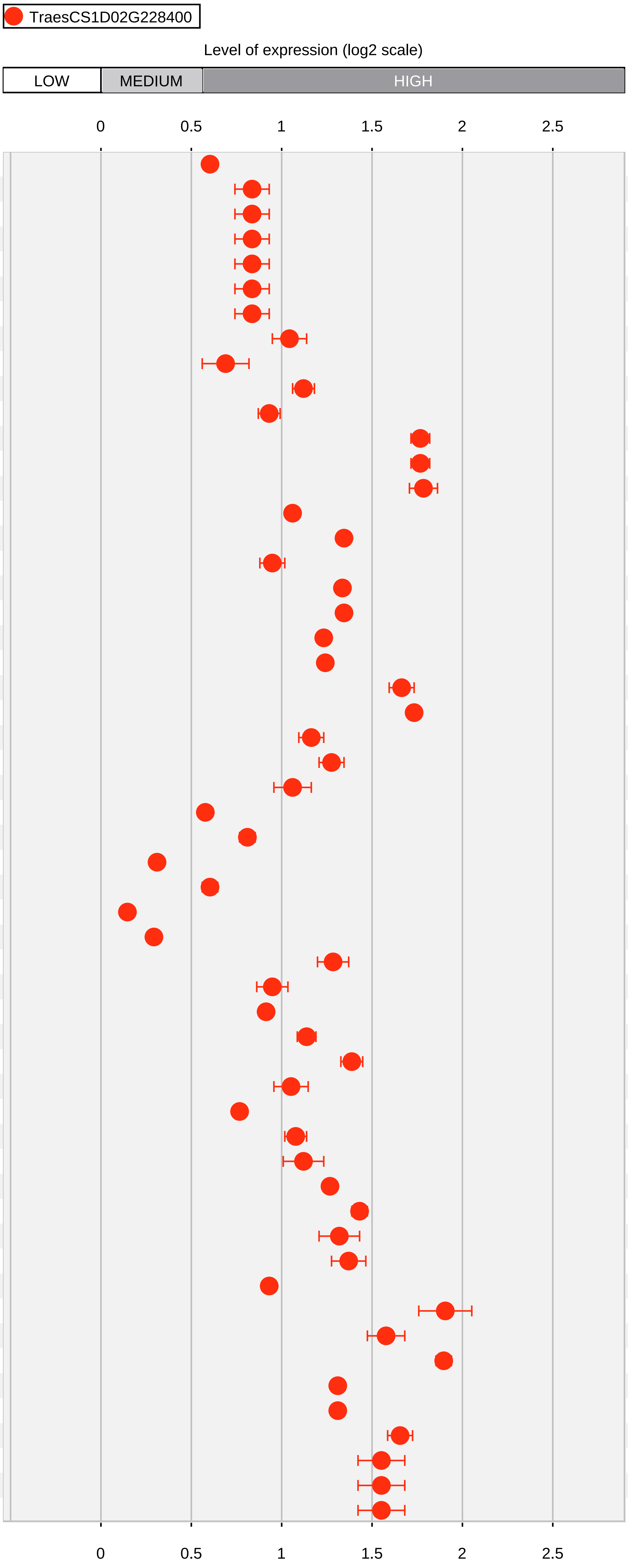

Triticum aestivum (55)

Anatomical part	samples	avg. expr.
callus	2	0.60
▼ cell culture / primary cell	15	0.84
▼ spike cell	15	0.84
▼ spikelet cell	15	0.84
▼ floret cell	15	0.84
▼ stamen cell	15	0.84
▼ anther cell	15	0.84
meiocyte	6	1.05
microspore	9	0.69
▼ seedling	47	1.12
coleoptile	34	0.93
▼ root	9	1.77
▼ radicle	9	1.77
radicle tip	6	1.78
▼ inflorescence	1055	1.06
▼ spike	660	1.35
rachis	28	0.95
▼ spikelet	260	1.34
▼ floret	170	1.34
▼ stamen	94	1.24
anther	79	1.24
▼ pistil	17	1.67
ovary	4	1.73
▼ lemma	11	1.17
awn	5	1.28
glume	6	1.06
▼ caryopsis	395	0.58
embryo	48	0.82
▼ endosperm	140	0.31
aleurone layer	21	0.61
starchy endosperm	35	0.15
endosperm transfer layer	19	0.29
▼ pericarp	13	1.28
outer pericarp	3	0.95
▼ shoot	1140	0.92
▼ culm (stem)	48	1.14
internode	8	1.39
peduncle	15	1.05
▼ leaf	739	0.77
blade (lamina)	35	1.07
sheath	15	1.12
▼ flag leaf	131	1.27
blade (lamina)	69	1.43
ligule	3	1.32
sheath	12	1.37
crown	86	0.93
▼ shoot apex	10	1.90
shoot apical meristem	6	1.58
axillary bud	6	1.90
▼ rhizome	199	1.31
▼ roots	199	1.31
nodal root	3	1.65
▼ unspecified root type	6	1.55
▼ root tip	6	1.55
root apical meristem	6	1.55

created with GENEVESTIGATOR
